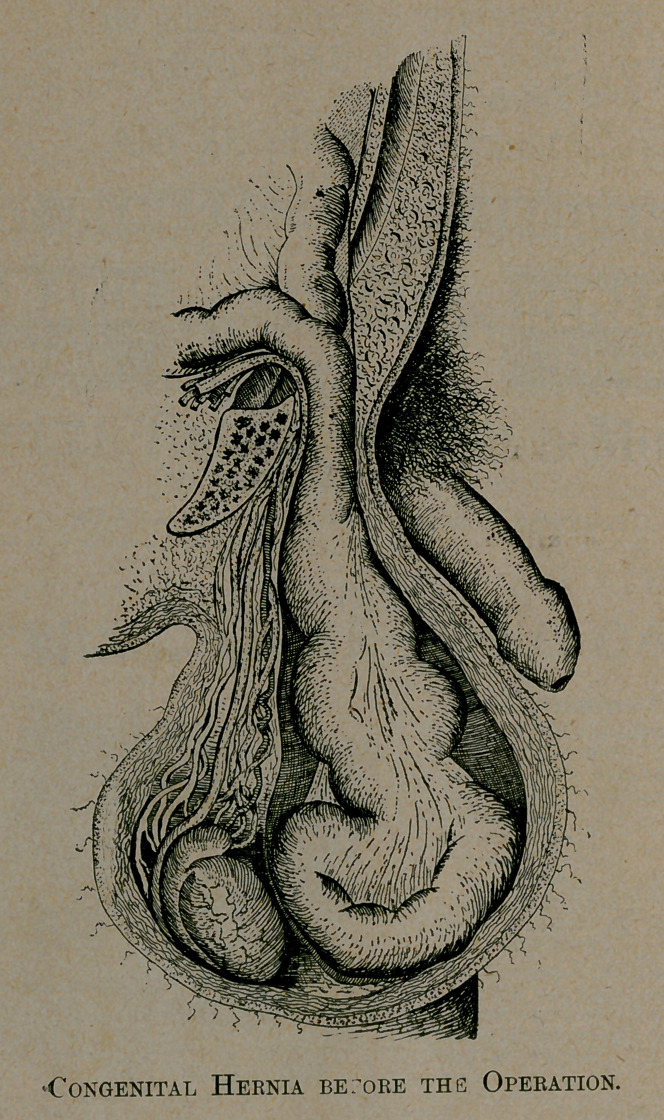# The Radical Cure of Hernia, Particularly with Children

**Published:** 1892-08

**Authors:** G. Felizet

**Affiliations:** Surgeon to Tenon Hospital, Paris, France


THE
### Southern Medical Record.
A MONTHLY JOURNAL OF MEDICINE AND SURGERY.

VOL. XXII. ATLANTA, GA., AUGUST, 1892. No. 8

### Original Articles.

### THE RADICAL CURE OF HERNIA, PARTICULARLY WITH CHILDREN.

BY DR. G. FELIZET, SURGEON TO TENON HOSPITAL, PARIS, FRANCE.

Translated by THOS. H. MANLEY, A. M., M. D., Visiting Surgeon to Harlem Hospital, New York.

### USE AND INDICATIONS FOR THE TRUSS.

For a surgical operation for hernia to merit the designation "radical cure," it will not suffice that the communication with the peritoneum is interrupted by ligation of the sac as high as possible, but the local conditions favoring its reproduction must be dissipated or removed before we are able to declare the result as a radical cure—*a hernia which does not exist and which cannot return.*

The sac in acquired hernia (we shall soon refer to congenital hernia) is the res lt of a s ipping rather than to the distension of the peritoneum. This sliding of the peritoneum operates in the way of protrusion when favored by a weak point.

This weak point manifests its defect as in the disproportion between abdominal effort and the resistance of the abdominal wall. The sac is the effect, the abdominal effort the occasion, and feeble point the cause of the production of the hernia.

To attack the sac only, is to expose the hernia to a speedy return.

It is to this weak point, then, that the surgeon must direct his efforts; to obliterate it and transform it, so that after operation it will be particularly solid and resistant. This result is within our power to obtain. Numerous cases of radical and definite cure of hernia may be seen to-day; and they would be more numerous, if operations were more frequently practiced under conditions favorable to rapidity of execution and final success.

In the surgery of infants, an operation long and tedious is always grave. Chloroform, shock and loss of blood are without doubt the elements of failure, rather than any act of the surgeon. But we cannot, nevertheless, reject an operation from this consideration alone, which in infants has given, and will give, brilliant results; more stable than in adults, in whom, in a general way, it may be preferable to employ the truss (bandage).

It is true that, as between an operation, uncertain in its results if any detail in treatment is neglected or fails, and which may be perilous and difficult in performance, easy and simple means of treatment are to be preferred, and hence, often the truss may be efficacious; at any rate, it is always free from danger, which may entail serious responsibility or occasion alarming symptoms, and one will usually prefer it.

It need not be repeated that the truss will cure the great majority of hernias in children; but it will not cure all, and it is for these *only* which the operation is specially recommended. The truss will cure recent, reducible hernias, when they are not voluminous and the patient not advanced in years.

But it should be remembered that its role in very early infancy is limited, by reason of the difficulties which present themselves in its application to the soft, tender tissues of the infant, which will not tolerate much pressure long, before they chafe and become rooded and besmeared, rendering any constant pressure inapplicable. For these reasons the truss must often be discarded in very early life.

The mechanism of cure in infantile hernia by the truss is simple. The intestine is retained in the abdomen; the opening which permits a passage ceases to be dilated, by repeated protrusions of the viscera, contracts; while the force which distends the abdominal walls increases with age, the relative dimensions of the ring diminish.

During this time, under the influence of pressure, the serous surfaces close about the sac, contracting reciprocally adhesions and intercept all communication with the cavity of the peritoneum in such a manner that the internal orifice is effaced, the tract reduced, the neck obliterated, and a recurrence of the hernia impossible.

The truss will sometimes overcome irreducible hernia; but the mechanism here is less simple and the results more doubtful. The pressure of the bulb will reduce, little by little, the intestine; crowding it upward and forcing it into the abdominal cavity, where it will be supported and retained with time, the adhesions between the intestine and the sac gradually giving away, provided they are not ancient, and have not acquired a tough, fibrous character. From time to time, in the treatment of this variety of hernia, the form and direction of the pilot must be changed, in order to follow and retain the receding bowel.

It is in these cases in which we see the best possible results in the hands of the prudent surgeon, who will not permit of any interference in the way of operation, until the truss has been patiently and carefully applied for a considerable period of time.

It is in this class of cases, too, in which when the truss has been long applied, no benefit will be derived; indeed, its continued application becomes dangerous, through the condition it has brought about in the hernia itself or in the patient. Not infrequently the truss will utterly fail to retain the hernia, and we will observe, instead of closing the tract through which the intestine escaped, it will widen and displace it. It is, under these circumstances, powerless to retain the protrusion, but powerful in the way of inducing strangulation, which it cannot obviate, but rather complicates.

In these cases, frequently, the pressure which is exercised on the cord leads to various affections—as pain and neuralgia of the cord, varicosity of the spermatic veins, hydrocele and orchitis.

A truss must be so constructed as to particularly fulfill two requirements: 1st—It must retain the hernia. 2d—It should be worn with comfort. A truss must supply these two factors or else be rejected altogether; for, as a rule, one should never wear a truss which improperly controls the protrusions or occasions suffering. We encounter hernias in which but one of these two difficulties obtain; and it is dependent rather on the consistence and shape of the pad, than on the peculiarity of the hernia itself. I am convinced that the double truss is the most valuable of all.

It would take too long here to describe, in detail, the principle on which the double truss acts when its *point d' oppin* is transferred partly from immediately over the internal ring to the entire circumference of the pelvis, which secures stability to the apparatus, and permits all movements and attitudes of the body. Besides, the frequent appearance of a hernia on the left side after the right side was cured, has for a long time led to the employment of the double truss as frequently in infants and children as in adults.

Although it is demonstrable and apparent when a truss is properly applied and it fails to serve any useful purpose, we must yet hope, for this in itself is not a justification for an immediate operation.

When we are assured it is free from undue pressure, no accident need be feared, and we may safely wait. We should always delay in certain cases of undescended testis (cryptorchidies), when the hernia is well advanced and the testicle is in the ring, restrained by the peritoneum and sac of the intestine. In this condition, neither truss nor operation—wait! Wait until the migration of the testicle and the full formation of the hernia, when we may proceed with mild, conservative methods, or by using the scalpel, according to indications presented. One should not delay, on the contrary, if the hernia is preceded or accompanied by the testicle; as, under the circumstances, it is certain to be the seat of accident, violent pains or strangulation itself. These are the conditions which often lead to strangulation, and the radical cure then is more than a complimentary and final; it is a kelotomy of urgence and necessity for the liberation or sacrifice of the ectopic testes.

The following was a case of the class under consideration: The patient, Alfred Legras, 14 years old, who was admitted with an intra-parietal ectopic testis with a hernia. He presented grave symptoms from a strangulated hernia and compression of the testicle. I performed an operation for his relief on the 25th of November, 1885, assisted by my *internes*, M. Breton *et* Marie Wilboutcheartch. A sac was discovered in which was found the strangulated bowel and a testis of normal size, which was easily drawn downward and maintained, thanks to the suture of the ring. The sac was not ligated. The youngster left the hospital on the 12th of January, 1886; but he was retained 15 days longer than usual on account of the nature of the operation. On the 20th of July, 1890, we found the testis well down externally and the inner aperture of the canal open. He now had no more pain in the testis nor threatening of the rupture's return; hence, the truss was entirely dispensed with.

If a descending testis contracts adhesions with the intestine, we should operate. In cases in which the testis has imperfectly descended, attended with no protrusion, by the time puberty is reached a practical cure may be effected by the normal lodgment of the testis and the gradual, but perfect, closure of the canal.

We had to do an immediate operation once, in a case in which the phenomena of compression of the testis and strangulation of intestine were well pronounced.

As a general rule, we advise delay in the ordinary hernia. A truss should be applied which is adaptable to the varying demands of the case, and worn until a cure is effected, or it would become a source of great discomfort. To determine how long it must be worn may be a matter of months or years. This is why, as a general rule, the radical cure of hernia by incision should not be recommended until after the fourth year, and if at this time, or even later, we are rewarded for our pains and patience by the retention of the protrusion, very much has been accomplished, and we have nothing to regret.

The wearing of the truss, even though not attended with the best results, always, nevertheless, causes a diminution in the size of the sac, a retraction of the neck and narrowing of the rings; conditions which favor the ulterior cure by radical intervention.

We have said not to operate under four or five years of age. "Not until five years of age," says M. Berger, "should the radical operation *ever* be undertaken." This limit of age is an effective rule of surgery. But it is a rule which has exceptions. For example, when one has to do a kelotomy for strangulation, we may effect a radical cure without compromising the life of the patient. On a case of this description we have operated as early as the eleventh month, and we have read of others.

However, we may be prepared for dangers and difficulties both before and after operation, when the hernias are of enormous size or augment rapidly in volume; when they contain many coils of intestine and constitute, in fact, a sort of accessory abdomen. Regardless of age, also, we must interfere as soon as possible when it appears that the hernia is rapidly increasing in size, though the truss be constantly worn. This year we operated on an infant eight months old, who had two large inguinal hernias descending as far down as the internal condyles of the femur. The operation was successful.

The operation for the radical cure of hernia on patients ranging from eight to eleven months of age is truly a surgical curiosity. But even with these successes, it is not to be generally recommended; though it is sometimes possible to interfere with impunity at a very early age.

INDICATIONS FOR THE RADICAL CURE OF HERNIA—RULES FOR OPERATIONS.

No one can upbraid us for hesitating to make fully known the advantages of the truss in effecting cures in early infancy and adolescence. We have endeavored to impress that an operation is not permissible or is inapplicable in every case of hernia, except those in which the truss is useless or intolerable; but we believe also that the radical cure of hernia by operation is nearly always free from dangers, if conducted according to the following indications :

1st. *Operate as rapidly as possible.*

2nd. *Have a careful regard for the nerves and vessels of the spermatic cord.*

3rd. *Carefully avoid bringing any septic matter in contact with the intestine, peritoneum or the wound.*

4th. *Be assured of complete obliteration of the intestinal canal.*

5th. *Establish a powerful septum, broad, strong and solid, at the point of depression of the internal orifice.*

After having performed operations for the radical cure on old men and adults, in an active service at Tenon Hospital, with infants and children more than twenty times for radical cure, we believe that we have devised a procedure which renders the operation short, simple, easy, harmless and definite, by a plan which is herewith described.

1st Indication—Operate as rapidly as possible.

It has been said that an operation unattended with difficulty was without value, as a radical cure must be a difficult operation; as if its ready performance was incompatible with rapid cure; as if the value of the procedure depended on something quite beyond the surgeon's art. The real difficulty lies in the varying methods and on individual, local conditions, the operation and the operator. But as a matter of fact, the result depends on the minute attention to detail and the most perfected methods.

Our work has for its object the presentation of a better *technique*, which will permit of a considerable reduction of the duration of operation. With equally satisfactory results, everyone will agree that a short operation is preferable to a prolonged and tedious one. A rapid operation has the advantage which less manipulation and division of structure implies, and the *minima* of the tissues are exposed to the irritating atmosphere. This is no indifferent advantage, when we are operating on infants or feeble subjects, incapable of enduring chloroform for an hour or more. The fear of a protracted operation has certainly prevented many surgeons of great authority from attempting the radical cure in cases often the most appropriate for this line of treatment. "It is imprudent," says M. Berger, "to extend an operation longer than the time necessary to expose the sac as far up as possible. We must endeavor to diminish that shock which may lead to mortal collapse and reduce to its shortest possible period that time during which the patient is submitted to the anæsthetizing agent. A practice of three years in the hospital of Bicetre has demonstrated to me that with the aged, afflicted with strangulated hernia, a cure is accomplished when an operation is rapidly conducted, and I have been led to renounce with them absolutely tentative methods for the radical cure."

When one can perform the operation for hernia in fifteen or twenty minutes, it must be seldom that there is any contra-indications. If one can diminish an operation two-thirds the time, we can comprehend why it might be possible at one *seance* to operate on two inguinal hernias as we have done on a patient with success, in 1887, and we also recently tried the same course on an infant with two enormous inguinal hernias, the report of which we have heretofore published. Our patient was but eight months old. We had intended to wait for several days before undertaking the second operation, but we completed both at the same time, and by a good stroke of fortune he recovered.

2nd Indication—Care of the vessels and nerves of the cord.

It is only necessary to see once for one's self, by the ordinary proceedings in an operation for the radical cure of a congenital or acquired hernia, to comprehend the extreme attention which must be given in order to avoid annulling the elements of the cord, its vessels, ducts and nerves. Very often it might be more correct to say rather the divided, scattered elements of the cord of which it is constituted. The vas deferens is easily made out in the adult, thanks to its consistence, yet one may wound it, as once happened to M. Lucas Championniere, and he was obliged by an accident of this sort to castrate.

The most trying to identify are the vessels; as they are lodged in a movable, soft mass, they are often very difficult to distinguish, and to ligate them would be to destroy the integrity of the testes. When in search for a spurting vessel, which we cannot readily control, however, we must apply the ligature, whatever it may be. And who knows but the secluded vessel is the spermatic vessel itself? This ligature secured amidst loose tissues, who knows how many nerve filaments may be engaged? But unfortunately, the compression of these nerve filaments may lead to serious consequences, to severe pain and grave nerve troubles of that same nature, which sometimes occasions the surgeon anxiety after castrations.

We believe that we have succeeded with a plan, by which in the division of the external arteries, in the division of the subcutaneous tissues, we have had to apply no ligatures in dissecting the sac and isolating the cord, and in congenital hernias as readily as in those acquired, they were made absolutely *a blanc.*

3rd Indication—To preserve the intestine, peritoneum and incision from all contact with septic infection.

We must have this in view not only in inguinal hernia, acquired or congenital, but hernia of every description. The preservation of the elements of the cord has been, till the present, our principal pre-occupation in the conduct of the operation.

We have now arrived to the neighborhood of the sac. What we shall say here will apply to other species of hernias, to crural hernia, certainly (we have operated on three of them by this means), and umbilical hernia. However, we have not yet tried it. The surgeon divides the tissues, ligates the arteries, if necessary, and preserves the elements of the cord without having yet disturbed it.

Without having lost any blood, the sac is reached and opened. Here many eventualities are to be observed:

1st. The hernia has not descended, the sac is empty, flat and imperceptible. This is an unlucky mishap, which we not infrequently encounter, and is very embarrassing. When the hernia is old and the patient an adult, we quickly reach it, and with the finger recognize the fibrous bed in which the sac is enclosed. With the infant the case is different; the sac is thin and fragile; it is neither recognized by its consistence nor its color. It is deeply buried, and quite lost in the adjacent tissues of the region.

There is but one rule in dealing with it—search. Search with prudence and the gentle use of the scalpel, aided by the forceps and the scissors to disassociate it from the connective tissue. Almost immediately under the point of the scalpel we see the thin, fragile layer, of a pearly pink color, so characteristic of the peritoneal investment. It is gradually stretched and a small opening made; its lips seized by the forceps and well opened.

In the third stage of the operation, it is the intestine with which we are chiefly occupied. The finger is now introduced and carried between the walls of the sac, above the ring, into the abdomen, in order to be assured that the gut has contracted no adhesions about the internal orifice. It is necessary in using the scalpel here, that caution is employed not to open the bowel, and to avoid this serious accident it is recommended after opening the sac to press the neck away from it with a small sponge, secured in a long forceps. When the pouch contains the intestine, it is not opened, and may be readily recognized by the color and consistence of its contents. No difficulty is now recognized, and with the division of the riveting tunics, the sac comes into view. The intestine reduced, it is yet pressed lightly so that the sac may be drawn downwards and out and piped in the opening with two forceps. The intestine having been completely returned, following the ordinary proceeding, the neck is pressed with a forceps moderately, a sponge applied high up and the dissection of the sac proceeded with.

If the intestine cannot be reduced, or reduced only in part, its detachment and complete liberation become a matter of difficulty and peril. If it appears that its adhesions are very firm and intimate, we act wisest by leaving it undisturbed. No danger is greater in attempting to separate the bowel, in certain cases, than is likely to follow by leaving it in position. If we remember that this dissection is accomplished on an irregular surface, changing and movable, without any firm support, we way appreciate the difficulties which attend it and which cannot be wholly avoided. It is in this class of cases we often prefer to simply open the sac and partly liberate it, rather than take chances or proceed in a laborious manner to completely detach it.

In these cases in which both sac and intestine are agglutinated, though dissection is possible, so much mutilation has been borne by the peritoneum that it is practically sacrificed, as the sac is bruised and torn, and is incapable often to serve as a guide for the final exclusion of the internal orifice of the ring.

Finally, when we meet with a congenital hernia with incomplete descent of the testis, in which a coil of intestine proceeds it, it is not enough to have recognized the sac, to have dissected it, to have re-entered the coil of herniated intestine; we must, besides, provide against the testis following it, because the testicle in rising upwards would be by its presence a permanent obstacle to the occlusion of the track, and there would remain a constant danger of strangulation at the internal orifice. It is then, with a view of securing perfectly a definite re-integration of the intestine above, and of permanently maintaining the testicle below, that we proceed to the fourth indication.

(*To be continued.*)

## Figures and Tables

**Figure f1:**